# Partial agenesis of the pectoralis major and minor muscle: A cadaveric case report

**DOI:** 10.1097/MD.0000000000039444

**Published:** 2024-09-13

**Authors:** Alejandro Bruna-Mejía, Mathias Orellana-Donoso, Pablo Nova-Baeza, Alejandra Suazo Santibañez, Gustavo Oyanedel-Amaro, Juan José Valenzuela-Fuenzalida

**Affiliations:** a Departamento de Cienciasy Geografía, Facultad de Ciencias Naturales y Exactas, Universidad de PlayaAncha, Valparaíso, Chile; b Escuela de Medicina, Universidad Finis Terrae, Santiago, Chile; c Department of Morphology, Faculty of Medicine, Universidad Andrés Bello, Santiago, Chile; d Department of Morphology and Function, Faculty of Health and Social Sciences, Universidad de las Américas, Santiago, Chile; e Facultad de Ciencias de la Salud, Universidad Autónoma de Chile, Santiago, Chile; f Department of Chemical and Biological Sciences, Faculty of Health Sciences, Universidad Bernardo O’Higgins, Santiago, Chile.

**Keywords:** congenital abnormalities, pectoralis muscles, Poland syndrome, Sprengel deformity, thoracic wall

## Abstract

**Rationale:**

The pectoralis major and minor muscles, located in the anterior chest wall, are crucial for upper limb movements.

**Patient concerns:**

Their nonsyndromic absence is rare but significant for surgical procedures involving the axillary and pectoral regions.

**Diagnoses:**

Ultrasound can confirm the diagnosis and delimit the extent of the muscular abnormality, detect abnormalities of the costal cartilages, among others.

**Interventions:**

This descriptive, cadaveric case report involves a formalin-fixed 57-year-old North American male, with no clinical or family history of similar conditions. The study was conducted at the Human Anatomy Laboratory of the School of Medicine of the Universidad Finis Terrae in Santiago, Chile, in August 2022.

**Outcomes:**

We present a cadaveric case of bilateral partial agenesis of the pectoralis muscles discovered during routine dissection. The pectoralis major muscle exhibited only the clavicular portion, with the sternocostal and abdominal portions absent and replaced by a thin layer of connective tissue bilaterally. The pectoralis minor muscle showed partial muscle fibers only in the most distal and inferior portions bilaterally.

**Lessons:**

This case report is significant due to the rarity of this condition without accompanying anatomical variations. Understanding this variant is valuable for clinical situations involving the shoulder and thorax region, such as trauma to the proximal third of the humerus, clavicular region, suprascapular region, and anterior chest wall. It may complicate conservative and/or surgical treatments due to different functional and irrigation patterns in the area and is also important for educating future professionals.

## 1. Introduction

The anterior thoracic wall, particularly the pectoralis major muscle (PMaj), exhibits several common variations. Despite its morphological variability, no classification system currently exists for the PMaj. These morphological variations may be associated with embryological development. The primary aim of this study is to create a new classification system for the PMaj in fetuses and to identify causes of the described morphological variations.^[1–5]^ The PMaj is a thick, fan-shaped muscle arising from the anterior surface of the sternal half of the clavicle (clavicular head); half the breadth of the anterior surface of the sternum down to the level of the 6th or 7th costal cartilage (sternal head); the 1st to the 7th costal cartilages (1st and 7th often omitted); the sternal end of the 6th rib; and the aponeurosis of the external oblique (rectus head). The clavicular fibers are usually separated from the sternal fibers by a slight cleft. The muscle converges to a flat tendon, approximately 5 cm wide, that is attached to the lateral lip of the intertubercular sulcus of the humerus and plays an essential role in upper limb movements, especially during adduction and medial rotation of the arm, participating synergistically with the posterior deltoid and posterior rotator cuff (infraspinatus and teres minor) during shoulder flexion from 0° to 90°, stabilizing the glenohumeral joint through the range of motion.^[6–8]^ The PMaj vascular supply is provided by 1 dominant vascular pedicle from the pectoral branch of the thoraco-acromial axis, supplemented by several smaller secondary segmental vessels from the deltoid and clavicular branches of the thoraco-acromial axis, and perforating branches of the internal thoracic arteries and superior and lateral thoracic arteries. The innervation is provided by the medial and lateral pectoral nerves. Fibres for the clavicular part are from C5 and C6; those for the sternocostal part are from C7, C8, and T1.^[6]^

Accessory muscles such as the sternalis muscle, pectoralis quartus muscle, chondroepitrochlearis muscle, and accessory PMaj muscle have been reported.^[1–3]^ Conversely, the most common variation of the PMaj muscle is the absence of the sternocostal head with associated hypertrophy of the clavicular head and absence of the pectoralis minor (PMin) muscle. Their non-syndromic absence is rare, with an incidence estimated at 1:11,000.^[9]^ Absence of the clavicular head alone is rare; although it may occur concomitantly with the occurrence of supernumerary muscles such as the sternoclavicularis or infraclavicularis.^[10,11]^ The absence of the pectoralis muscles is usually partial and unilateral and can be part of a syndrome, such as Poland syndrome, which is accompanied by other associated anomalies, such as ipsilateral syndactyly (webbed, short, or missing fingers/toes or hypoplasia of the hand).^[12,13]^ It is referred to as Sprengel deformity when resulting from hypoplasia of the serratus anterior muscle, characterized by congenital elevation of the scapula, the most common congenital abnormality of the shoulder girdle.^[14,15]^ Generally, the absence of the sternocostal portion of the PMaj muscle, with or without the absence of the PMin muscle, is the most frequent,^[9]^ while the absence of the sternocostal part of the PMaj muscle alone is the least frequent.^[16]^ Associated anomalies can include deficiencies of the chest, breast, nipple, ribs, costal cartilage, axillary hair, and sweat glands (on the affected side), and scoliosis can also be present.^[17–19]^

The PMin muscle is a thin, triangular muscle lying posterior (deep) to the PMaj. It arises from the upper margins and outer surfaces of the 3rd to 5th ribs (frequently, 2nd to 4th), near their cartilages, and from the fascia over the adjoining external intercostal muscles. Its fibers ascend laterally under cover of the PMaj, converging in a flat tendon that is attached to the medial border and upper surface of the coracoid process of the scapula, and laterally contributing to form the coracoacromial ligament. Its vascular and nerve supply is provided by the pectoral and deltoid branches of the thoraco-acromial and superior and lateral thoracic arteries, and it is innervated by branches of the medial and lateral pectoral nerves, coming from the anterior rami of C5, C6, C7, C8, and T1 spinal nerves.^[6]^ Anatomical PMin variants, which can present as variations of the proximal insertion, different from the “typical” origin of the PMin muscle, are ribs 3 through 5, the most common being ribs 2 through 4.^[6,18–21]^ Non-syndromic complete or bilateral absence is rare, affecting about 0.01% of the worldwide population.^[22,23]^ Additionally, there have been only 4 cases of non-syndromic unilateral complete absence of the pectoralis muscles reported in China. The first 3 cases were found in patients,^[24–26]^ with one of them associated with a pterygoid anterior axillary fold,^[24]^ and the 4th case was found during a routine cadaver dissection.^[27,28]^ Patients with an absent PMaj and PMin, despite usually having a 20% to 30% decrease in horizontal shoulder adduction strength, retain full function, and experience no decrease in arm internal rotation strength or other athletic restrictions, due to compensation provided by neighboring muscles such as the subscapularis and anterior serratus.^[10,26]^

## 2. Case report

**Figure 1.**
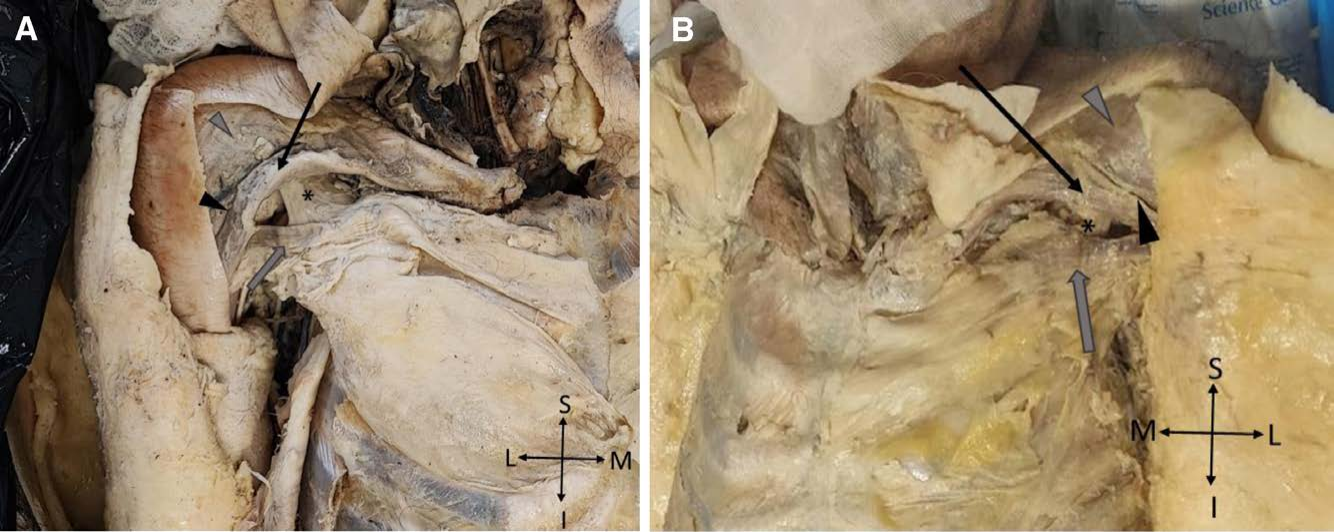
Dissection of the superficial anterior thoracoappendicular muscles. (A) A dissection of the right pectoral region showing the clavicular (black arrow) and sternocostal (gray arrow) portions of the PMaj muscle, between the 2 portions the PMin (*) can be seen, and the deltopectoral sulcus (black arrow head) between the sternocostal portion of the PMaj and the deltoid’s clavicular portion. (B) A dissection of the left pectoral region showing the clavicular (black arrow) and sternocostal (gray arrow) portions of the left PMaj muscle, the PMin (*) between them, and between the clavicular portion of the PMaj and the left deltoid’s clavicular portion (gray arrow head) the deltopectoral sulcus is shown (black arrow head).

**Figure 2.**
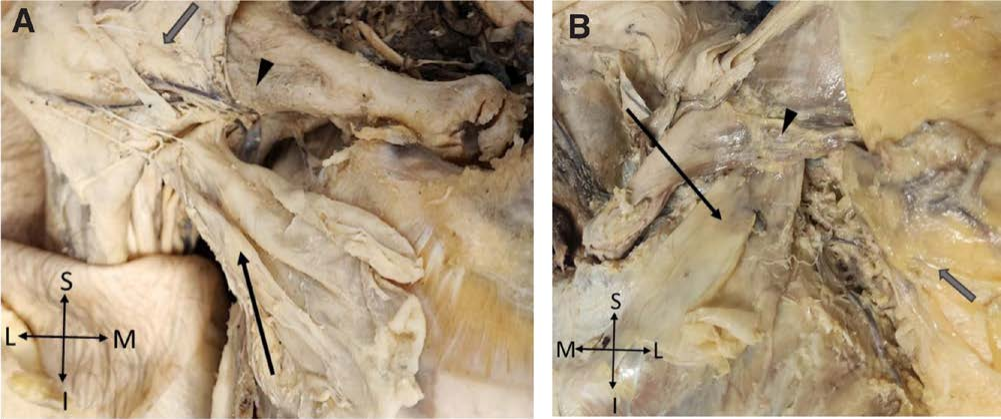
Dissection of the deep anterior thoracoappendicular muscles. (A) A dissection of the deep right pectoral region and (B) a dissection of the left pectoral region showing the clavicular portion (black arrow head), the sternoclavicular connective tissue (gray arrow) portion of the PMaj, and the PMin muscle blending with connective tissue before attaching to the ribs (black arrow).

**Figure 3.**
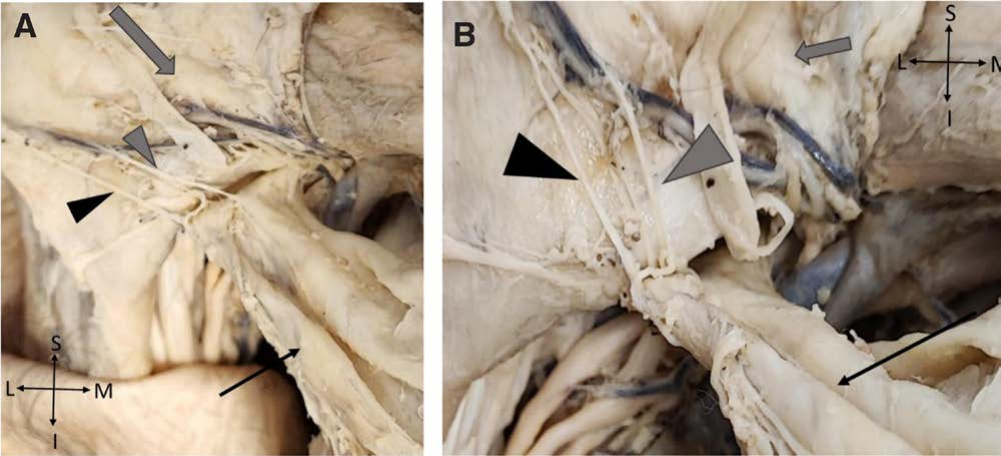
Right pectoral region (A and B) where the PMaj (gray arrow) was removed so it can be seen the PMin (black arrow) muscle and the lateral (black arrow head) and medial (gray arrow head) pectoral nerves.

This descriptive cadaveric case report presents a comprehensive examination of a 57-year-old North American male, who died of myocardial infarction without any preceding clinical or familial history suggestive of similar conditions. The specimen, preserved in formalin, was meticulously studied at the Human Anatomy Laboratory, School of Medicine, Universidad Finis Terrae, Santiago, Chile, in August 2022, providing a unique opportunity for in-depth analysis and observation. The institution has authorization to conduct teaching and research without requiring additional ethical approval, as this is addressed when specimens enter the anatomy laboratories under resolution code S:49-2022-2094. Furthermore, it is important to note that this study did not involve human participants or live animals. The anterior thoracic wall had no visible abnormalities, but during dissection, a bilateral partial agenesis of the PMaj and the PMin was observed. The dissection was carried out by an anatomist with more than 46 years of dissection experience. The tools used included hemostatic forceps, a scalpel handle with a metal handle and a 22 blade, a tissue forceps with 1X2 teeth, and Kelly hemostats. During the dissection, only the clavicular fibers of the PMaj were formed, attaching along the anterior border of the clavicle, while the sternal and costal portions were not fully present, and the abdominal portion was absent bilaterally. Instead, arranged connective tissue was observed on the left PMaj 13 cm from the humeral insertion and on the right PMaj 10 cm from the humeral insertion. Laterally, it attaches to the anterior aspect of the humerus on the lateral lip of the intertubercular sulcus through a 3 cm wide bilaminar band, with an anterior lamina formed by the clavicular fibers that prolong into the distal tendon of the deltoid, and a posterior one formed by the convergence of the sternocostal fibers. Additionally, the PMin was not fully formed bilaterally. The left PMin had a length of 13 cm, with muscle fibers running from the coracoid process but blending with connective tissue at approximately 2.5 cm, which attached to the inferior border of the 4th rib. The superior portion was purely connective tissue, running from the coracoid process to the 2nd to 4th ribs. The right PMin had a length of 15 cm, running from the coracoid process to the 1st to 4th ribs, with muscle fibers distinguishable in the inferior border, blending with connective tissue at 9 cm from the distal insertion. The PMaj was innervated by both the medial and lateral pectoral nerves bilaterally, while the PMin was innervated only by the medial pectoral nerve bilaterally, forming the asa pectoralis superficially around the PMin muscle. No other incidental abnormality of the thoracic wall, upper limb, or other medical diagnosis was found (Figs. 1–3 and Appendix 1, Supplemental Digital Content (Pectoral Agenesis, http://links.lww.com/MD/N449)).

## 3. Discussion

During a routine dissection, we found a PMaj muscle with bilateral partial agenesis, presenting only its clavicular portion. This condition has been associated with Poland syndrome, characterized by partial or total absence of unilateral or bilateral pectoral muscles, often accompanied by other anomalies such as absence of the nipple, absence or reduction of the mammary gland, and syndactyly ipsilateral to the agenesis.^[29–31]^ It is also associated with Sprengel deformity, characterized by hypoplasia of the serratus anterior muscle and congenital elevation of the scapula.^[14,15]^ In this case report, the cadaver presented partial agenesis of both the PMaj and PMin but without abnormalities related to Poland syndrome or Sprengel deformity.^[19]^ This variant is described as an accidental occurrence without genetic predisposition.^[32–38]^

Skeletal muscle development originates from the mesoderm, particularly the paraxial mesoderm, organized alongside the neural tube in somites during the 4th to 8th weeks of embryonic development. The somites contain 2 subpopulations of cells: the dorsolateral dermomyotome and the ventromedial sclerotome. The dermomyotome differentiates into skeletal muscle, with the anterior embryonic muscles formed from hypomere cells, potentially leading to anatomical variations such as those observed in the pectoralis muscles. PMin’s inferior origin variations are rare, with cases documented where the muscle originates from only the 5th rib or the 6th rib.^[21]^ Proximal attachments from ribs 1 to 7 have also been reported but are generally clinically silent. Regarding distal insertion variations, LeDouble (1897) classified PMin distal insertions into 3 types: Type I, where the deep part of the tendon attaches to the coracoid process in a standard fashion, but the superficial part passes over it to a more proximal structure; Type II, where most fibers attach to the coracoid process with a few passing over it; and Type III, where the tendon passes over the coracoid process as a unit without attaching, often separated by a bursa.^[35]^ Type III variations are often concomitant with the absence of the coracohumeral ligament.

In a study by Tubbs et al (2005), a left PMin originated at the 3rd through 5th costochondral junctions, traveling over the coracoid process without attaching and inserting directly into the fibrous joint capsule; in contrast with the right shoulder that showed no musculoskeletal abnormalities. A study by Lee et al (2010) found that in a retrospective MRI study of 355 shoulders, 5 cases (1.5%) had a PMin tendon inserting into the joint capsule with associated absence of the coracohumeral ligament. Additionally, 3 of these 5 patients had superior labrum anterior to posterior lesions, suggesting that anomalous insertions of the PMin tendon to the glenohumeral joint might be related to the absence of the coracohumeral ligament and could contribute to superior labrum anterior to posterior lesions. Patients with LeDouble Type III variations and absence of the coracoacromial ligament might be at higher risk for such injuries.^[37]^ Dayal et al (2014) suggested that this variant could be a type of Poland syndrome, but we believe it should not be classified as such due to the absence of specific characteristics.^[39,40]^ Yuan et al (2018) described the absence of the PMaj muscle as a non-syndromic congenital variant with significant implications. They found that Poland syndrome, in some cases, is associated with leukemia and kidney disorders, suggesting that agenesis of the PMaj muscle could worsen the prognosis in the presence of infection and chronic disease. Follow-up should be conducted based on these clinical pictures.^[34,41,42]^ However, there is insufficient evidence to support this theory, highlighting the need for future studies to confirm or refute these findings.

## 4. Limitations

Limitations of this case report include the limited information regarding the subject’s clinical history.

## 5. Conclusion

The agenesis of the PMaj muscle is a rare anatomical variant of the anterior thoracic region, which does not cause significant biomechanical alterations in shoulder functionality or the thoracic region. Although it presents characteristics of Poland syndrome or Sprengel deformity, it should not be classified as such due to the absence of all specific characteristics. Knowledge of this variant is useful for clinical aspects related to the shoulder and thoracic region and for teaching future professionals.

## Acknowledgments

As a research team, we thank our colleagues who made it possible to finish this research work.

## Author contributions

**Conceptualization:** Alejandro Bruna-Mejía, Pablo Nova-Baeza, Juan José Valenzuela-Fuenzalida.

**Data curation:** Mathias Orellana-Donoso, Gustavo Oyanedel-Amaro, Juan José Valenzuela-Fuenzalida.

**Formal analysis:** Mathias Orellana-Donoso.

**Funding acquisition:** Alejandro Bruna-Mejía, Alejandra Suazo Santibañez.

**Investigation:** Pablo Nova-Baeza, Alejandra Suazo Santibañez, Gustavo Oyanedel-Amaro.

**Methodology:** Mathias Orellana-Donoso, Juan José Valenzuela-Fuenzalida.

**Project administration:** Mathias Orellana-Donoso, Pablo Nova-Baeza, Alejandra Suazo Santibañez, Juan José Valenzuela-Fuenzalida.

**Resources:** Mathias Orellana-Donoso.

**Software:** Alejandra Suazo Santibañez.

**Visualization:** Alejandro Bruna-Mejía, Pablo Nova-Baeza, Gustavo Oyanedel-Amaro.

**Writing – original draft:** Alejandro Bruna-Mejía, Alejandra Suazo Santibañez, Gustavo Oyanedel-Amaro, Juan José Valenzuela-Fuenzalida

**Writing – review & editing:** Alejandro Bruna-Mejía.

## Supplementary Material


